# A low grade fibromyxoid sarcoma originating from the masseter muscle: a case report

**DOI:** 10.1186/s13256-015-0658-9

**Published:** 2015-08-21

**Authors:** Eun Jung Lee, Hye Jin Hwang, Hyung Kwon Byeon, Heae Surng Park, Hong-Shik Choi

**Affiliations:** 1 Department of Otorhinolaryngology, Gangnam Severance Hospital, Yonsei University College of Medicine, 211 Eonju-ro, Gangnam-gu, 135-720, Seoul, Korea; 2 Department of Pathology, Gangnam Severance Hospital, Yonsei University College of Medicine, 211 Eonju-ro, Gangnam-gu, 135-720, Seoul, Korea

**Keywords:** Fibromyxoid sarcoma, Head and neck, Masseter

## Abstract

**Introduction:**

Low grade fibromyxoid sarcoma is a distinctive variant of fibrosarcoma. We recently experienced a rare case of low grade fibromyxoid sarcoma arising in the masseter muscle.

**Case presentation:**

A 63-year-old Korean woman with a large growing mass in her right parotid gland area for 1 year visited our clinic. Complete removal of the tumor was achieved by parotidectomy with facial nerve preservation. The tumor measured over 4cm with pathologic findings compatible with low grade fibromyxoid sarcoma.

**Conclusions:**

Low grade fibromyxoid sarcoma is an extremely rare tumor, and report of the present case is noteworthy since it represents a rare localization of low grade fibromyxoid sarcoma in the head and neck. Close follow up on a long-term basis is considered necessary because of its high potential to metastasize.

## Introduction

Low grade fibromyxoid sarcoma is a cytologically bland malignant neoplasm with alternating fibrous and myxoid stroma of low grade and malignant potential [1]. Low grade fibromyxoid sarcoma is considered a rare soft tissue tumor with a high metastasizing potential, despite its benign histologic appearance. Low grade fibromyxoid sarcomas represent approximately 10% of soft tissue sarcomas and are rarely found in the head and neck region. The most common locations are the extremities, trunk, chest and soft tissues.

There have been only five reported cases of low grade fibromyxoid sarcoma in the head and neck [2]. Low grade fibromyxoid sarcoma originating from the masseter muscle has never been reported before. We recently experienced a rare case of low grade fibromyxoid sarcoma in the masseter muscle. Due to the relative rarity of low grade fibromyxoid sarcomas, there is no consensus regarding treatment and postoperative follow-up recommendations. Nevertheless, the low grade fibromyxoid sarcoma has potential for late local recurrence and distant metastasis [3]. In order to detect possible metastasis in advance it is important to inform the patients about the longstanding metastatic potential of the disease and recommend prolonged follow-up.

## Case presentation

**Fig. 1:**
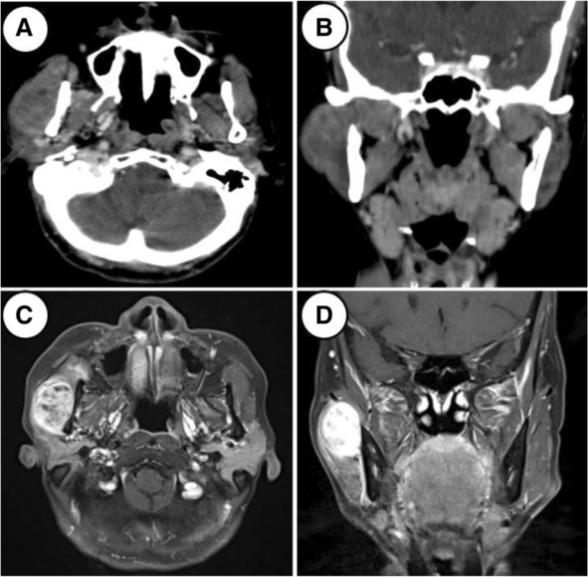
Axial (**a**) and coronal (**b**) images of neck computed tomography. The mass shows a heterogeneous density on right parotid gland, with no erosion of adjacent structure. Axial (**c**) and coronal (**d**) images of neck magnetic resonance imaging. A 3.5×2.0cm-sized, irregular internal gadolinium-enhanced mass can be seen between the right parotid area extending to masseter muscle. The lesion is relatively well demarcated with smooth margins

A 63-year-old Korean woman who had no medical history presented with swelling at her right parotid gland area that had persisted for 1 year. No abnormal findings were found elsewhere, and the other laboratory tests showed normal. A physical examination revealed a fixed non-tender round-shaped mass at her right parotid gland area. Images of neck computed tomography (CT) revealed a 3.2cm-sized heterogeneously enhancing mass which was located lateral to the mandibular rami and next to her right masseter muscle (Fig. 1a, b). Neck magnetic resonance imaging (MRI) showed a well-demarcated, 2.5×3.0cm-sized, gadolinium-enhanced, and fungating mass with internal hemorrhage and increased vascularity (Fig. 1c, d). Ultrasonography showed a heterogeneous hypoechoic mass in her right parotid gland suspicious for parotid gland tumor, and a fine-needle aspiration biopsy showed a few fragments of relatively short spindled cells in myxoid and bloody background. Cytologists also suggested that we rule out pleomorphic adenoma.

**Fig. 2:**
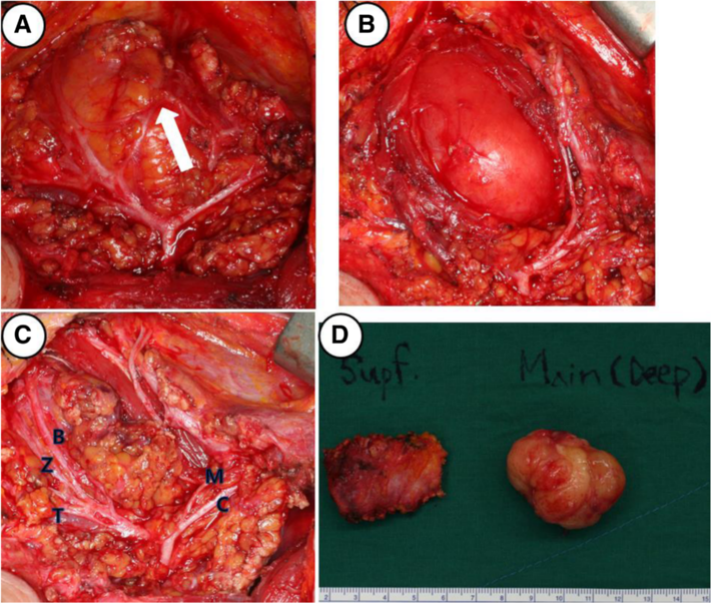
Intraoperative findings. Communicating branch (*white arrow*) between marginal branch and buccal branch was noted and sacrificed to remove complete mass excision (**a**). The main mass was located adjacent to masseter muscle in the deep lobe of parotid gland (**b**). All facial nerve branches were preserved: temporal (*T*), zygomatic (*Z*), buccal (*B*), marginal mandibular (*M*), and cervical branches (*C*) (**c**). Gross finding of main mass showed well-demarcated margin (**d**) margin (Supf. = superficial lobe of parotid gland, Main (Deep) = deep lobe of parotid gland)

Superficial parotidectomy was done with modified facelift incision. The main mass adjacent to her masseter muscle was located at the anterior aspect of her parotid gland (Fig. 2a). Pes anserinus and all branches of her facial nerve were identified and preserved (Fig. 2b, c). The retrieved specimen revealed a 4cm-sized mass (Fig. 2d).

**Fig. 3:**
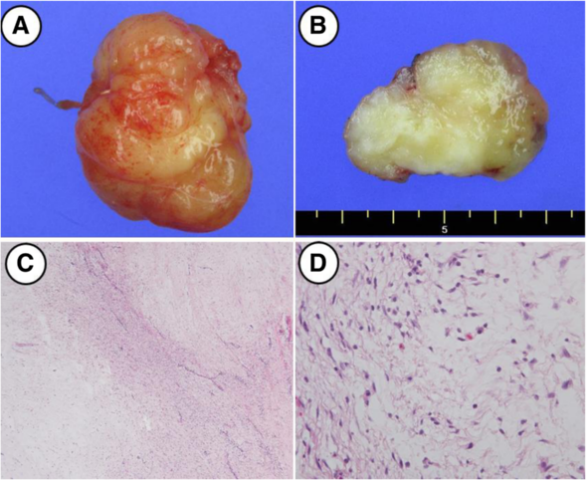
Gross finding and histopathological view. The main mass showed well-margined shape (**a**). Cut section showed well-demarcated, yellow-whitish mass with focal glistening area (**b**). Low power view showed tumor with alternating fibrous and myxoid areas on hematoxylin and eosin ×40 (**c**). High power view showed spindle tumor cells with minimal atypia on hematoxylin and eosin ×400 (**d**)

On macroscopic examination, the tumor was well-demarcated and had a yellow-white appearance with focal glistening areas on a cut section (Fig. 3a). On microscopic examination, the tumor had alternating fibrous (cellular) and myxoid (hypocellular) areas (Fig. 3b). The tumor cells were spindle and bland-looking with rare mitosis (Fig. 3c, d). The histopathologic findings of this tumor were compatible with low grade fibromyxoid sarcoma. On postoperative examination, weakness of her marginal facial nerve was noted; however, it was completely resolved on serial close follow up. She was disease-free with no evidence of recurrence after 1-year follow up.

## Discussion

Low grade fibromyxoid sarcoma was first described by Evans in 1987 and is currently classified as a malignant fibroblastic/myofibroblastic tumor by the World Health Organization [1]. These sarcomas typically affect mid-adults and arise most commonly in the proximal extremities and trunk; however, low grade fibromyxoid sarcomas can also arise at the head and neck region [2]. There are two reported cases which locally originated from the glands of head and neck: one case from the thyroid gland and one case from the parotid gland, the latter being a giant (12×8cm) primary low grade fibromyxoid sarcoma originating from the parotid gland and being completely removed with sacrifice of the facial nerve [4, 5].

This is the first reported case of a low grade fibromyxoid sarcoma confined to the soft tissue of the masseter muscle. The diagnosis of low grade fibromyxoid sarcoma was confirmed by the characteristic histological appearance with immunohistochemical staining [6, 7]. On histological examination, low grade fibromyxoid sarcoma shows alternating fibrous and myxoid areas. Also, the surrounding cells are more crowded, round or ovoid, and blend imperceptibly with the surrounding spindle-shaped cells. On immunohistochemical examination, it usually shows intense immunostaining for vimentin, P53, bcl-2 and smooth muscle actin (SMA) and negative for cytokeratin (CK)-19, galectin-3, and calcitonin; this finding may help distinguish low grade fibromyxoid sarcoma from other sarcomas [7, 8]. In the present case, preoperative aspiration cytology showed a few small clusters and fragments of relatively bland-looking short spindled cells in myxoid and bloody background. The possibility of pleomorphic adenoma could not be ruled out. However, histologic findings of hematoxylin and eosin staining and immunohistochemistry were compatible with those of low grade fibromyxoid sarcoma. The main concern for differential diagnosis would be myxofibrosarcoma. Myxofibrosarcoma commonly arises in the subcutaneous tissue of the elderly, while low grade fibromyxoid sarcoma typically arises in the skeletal muscle of young patients. On histological examination, myxofibrosarcoma is uniformly myxoid and has a greater degree of atypia than low grade fibromyxoid sarcoma.

Mucin 4 (MUC4) is a highly sensitive and specific immunostaining marker for low grade fibromyxoid sarcoma [9]. In addition, low grade fibromyxoid sarcomas have one of two distinct chromosomal translocations – t(7;16)(q34;p11) or t(11;16)(p11;p11) – that results in the fused in sarcoma (*FUS*)*-CREB3L2* and *FUS-CREB3L1* gene fusions [10]. Therefore, a specific immunostaining marker such as *FUS* gene arrangement will also be useful for diagnosing low grade fibromyxoid sarcomas. However, generally, low grade fibromyxoid sarcomas can be easily distinguished from other sarcomas on the basis of histopathological findings. A fluorescent *in situ* hybridization (FISH) probe and detection of specific immunostaining marker such as MUC4 would not be practical in terms of cost effectiveness. Therefore utilizing FISH to detect a *FUS* gene rearrangement or *MUC4* gene is an ancillary method for diagnosing low grade fibromyxoid sarcomas.

Imaging features of low grade fibromyxoid sarcoma are generally nonspecific [11]. Benign sarcomas are usually well encapsulated, whereas malignant sarcomas may show extensive infiltration. Bone destruction can occur secondary to pressure erosion and such a finding does not necessarily indicate malignancy. In the present case, a fungating mass with internal hemorrhage and increased vascularity was noted on neck MRI, but these findings were not specific for low grade fibromyxoid sarcoma. In fact, preoperative imagings suggested the presence of a hemangioma rather than the low grade fibromyxoid sarcoma. On intraoperative examination, the tumor main mass was well marginated and complete removal of the mass was relatively easy. Pathologically enlarged lymph nodes were not identified in the operation field.

Complete surgical excision with a safety margin is the mainstay of treatment for patients with low grade fibromyxoid sarcoma [3, 6]. The role of radiotherapy and chemotherapy remains controversial; however, radiotherapy has been indicated in cases in which the tumor cannot be completely resected [3]. In the present case, we were unable to predict the presence of a malignant tumor on the basis of preoperative imaging studies, and we planned complete surgical removal since the margins were noted to be smooth and relatively clear. The surgical margins were defined according to the principles of Enneking. Enneking *et al*. reported the 5-year overall survival rate following surgery for low grade fibromyxoid sarcoma is over 90%, with a more favorable prognosis associated with smaller tumors [12]. Local recurrences of low grade fibromyxoid sarcoma have been reported from a few months to 15 years after initial treatment, while distant spread has occurred from 0 to 45 years after surgery. Radiotherapy or chemotherapy may be necessary for patients whose tumors recur locally or spread to distant sites.

Low grade fibromyxoid sarcoma is a slow-growing tumor, but this cancer has a tendency to recur. It has been known to spread to distant organs many years after surgery; therefore, close serial follow up on a long-term basis would be required to prevent local or distant metastasis due to the tendency to recur after initial treatment.

## Conclusions

This is the first report in the medical literature of a low grade fibromyxoid sarcoma arising from the masseter muscle. Although low grade fibromyxoid sarcoma has the possibility of distant metastasis, it is likely that this patient may have a favorable prognosis with long-term close follow up because the tumor was solitary, well capsulated, confined to the localized area, and did not show severe mitosis in histology.

## Consent

Written informed consent was obtained from the patient for publication of this case report and any accompanying images. A copy of the written consent is available for review by the Editor-in-Chief of this journal.

## Abbreviations

FISH: Fluorescent *in situ* hybridization
*FUS*: Fused in sarcoma
MRI: Magnetic resonance imaging
MUC4: Mucin 4
